# Multitask knowledge-primed neural network for predicting missing metadata and host phenotype based on human microbiome

**DOI:** 10.1093/bioadv/vbae203

**Published:** 2024-12-13

**Authors:** Mahsa Monshizadeh, Yuhui Hong, Yuzhen Ye

**Affiliations:** Computer Science Department, Indiana University, Bloomington, IN 47408, United States; Computer Science Department, Indiana University, Bloomington, IN 47408, United States; Computer Science Department, Indiana University, Bloomington, IN 47408, United States

## Abstract

**Motivation:**

Microbial signatures in the human microbiome are closely associated with various human diseases, driving the development of machine learning models for microbiome-based disease prediction. Despite progress, challenges remain in enhancing prediction accuracy, generalizability, and interpretability. Confounding factors, such as host’s gender, age, and body mass index, significantly influence the human microbiome, complicating microbiome-based predictions.

**Results:**

To address these challenges, we developed MicroKPNN-MT, a unified model for predicting human phenotype based on microbiome data, as well as additional metadata like age and gender. This model builds on our earlier MicroKPNN framework, which incorporates prior knowledge of microbial species into neural networks to enhance prediction accuracy and interpretability. In MicroKPNN-MT, metadata, when available, serves as additional input features for prediction. Otherwise, the model predicts metadata from microbiome data using additional decoders. We applied MicroKPNN-MT to microbiome data collected in mBodyMap, covering healthy individuals and 25 different diseases, and demonstrated its potential as a predictive tool for multiple diseases, which at the same time provided predictions for the missing metadata. Our results showed that incorporating real or predicted metadata helped improve the accuracy of disease predictions, and more importantly, helped improve the generalizability of the predictive models.

**Availability and implementation:**

https://github.com/mgtools/MicroKPNN-MT.

## 1 Introduction

The human microbiome is a sophisticated ecosystem encompassing trillions of microorganisms, demonstrating a crucial impact on human health and diseases (Hou *et al.* 2022). This intricate microbial community is distributed across various body sites, including the skin, oral cavity, respiratory tract, gastrointestinal tract, urinary tract, and reproductive tract (Huttenhower *et al.* 2012). It is involved in diverse physiological processes, ranging from digestion and immunity to metabolism and brain function. Maintaining the delicate balance of the microbiome is essential, as disruptions can result in dysbiosis, associated with a broad spectrum of diseases, including inflammatory conditions, obesity, diabetes, and depression (Wilkins *et al.* 2019; Hou *et al.* 2022). Consequently, the exploration of the human microbiome has emerged as a rapidly expanding field of research, holding substantial promise for advancements in disease diagnosis and treatment, among others. Metagenomic data analysis of the human microbiome has become a widely adopted strategy for exploring its impact on human health and disease. The link between the human microbiome and human health has generated considerable interest in using microbiome data for disease diagnosis and personalized medicine (Laterza and Mignini 2022).

Many different predictive models have been developed for microbiome-based disease prediction (Marcos-Zambrano *et al.* 2021; Liu *et al.* 2024). The models vary in the types of inputs they take (species profiles, functional profiles, or both), Machine Learning (ML) and AI algorithms, and the prediction targets (single-disease or multi-disease) (Le Goallec *et al.* 2020; Wirbel *et al.* 2021). These models have various prediction accuracy and interpretability (some are black box, whereas others are more interpretable). Early predictive models were based on conventional ML/AI algorithms, and more recently, deep learning methods including various autoencoders were also exploited for learning the representation of quantitative microbiome profiles in a lower dimensional latent space for building predictive models. Examples include DeepMicro (Oh and Zhang 2020), Ph-CNN (Fioravanti *et al.* 2018), PopPhy-CNN (Reiman *et al.* 2020), and EPCNN (Chen *et al.* 2022). We have previously developed MicroKPNN (Monshizadeh and Ye 2024) for predicting human health status based on microbiome data, aiming to improve the accuracy of prediction and at the same time provide good explainability of the predictions. MicroKPNN incorporates multiple microbial relationships (metabolic, phylogenetic, and community) in the model to improve the performance of microbiome-based prediction and interpretability of the models. The model enables the examination of the importance of different input species and possible explanations (through the hidden nodes that have biological meaning). MicroKPNN achieved encouraging performance when tested on seven gut microbiome datasets involving five different human diseases.

Predictions of human diseases that could benefit from using microbiome include liver diseases (Caussy *et al.* 2019), respiratory diseases (asthma (Bannier *et al.* 2020) and COPD (Bowerman *et al.* 2020)), diabetes (T1D (Zheng *et al.* 2018) and T2D (Iatcu *et al.* 2021)), inflammatory bowel disease (Mills *et al.* 2022), and neuropsychiatric conditions (Liu *et al.* 2024). A growing body of work has also demonstrated the possibility of using the gut microbiome for the prediction of future onset of diseases. Examples include a longitudinal study showing that the gut microbiome at 1 year of age can distinguish individuals who develop future T1D up to 20 years later from those who do not (Bélteky *et al.* 2023), and another study that shows baseline gut microbiome is associated with new-onset T2D in up to 18 years (Ruuskanen *et al.* 2022).

Various confounding factors affect human microbiota, and the factors include sex, diet, race, medications, host’s genetic variation, and so on. In addition, there are technical artifacts that could complicate the use of microbiome data (Puschhof and Elinav 2023). Animal and human studies have shown sex differences in gut microbiota, and different mechanisms were suggested (Valeri and Endres 2021; Kim 2022). Results from an animal experiment involving microbiota transfer suggested that the microbiota-independent gender differences in the immune system select a gender-specific gut microbiota composition, which in turn further contributes to gender differences in the immune system (Fransen *et al.* 2017). Sex hormones are a potent driver of differences in the microbiome, and other factors (diets, antibiotics and environment) impact gut microbiota in a sex-dependent manner (Valeri and Endres 2021). A joint analysis (Blekhman *et al.* 2015) of the composition of the human microbiome and host genetic variation revealed significant associations between host genetic variation and microbiome composition, and these associations are found to be driven by host genetic variation in immunity-related pathways and genes associated with microbiome-related complex diseases including inflammatory bowel disease and obesity-related disorders.

In addition to models that have been developed to utilize microbiome data for human disease prediction, microbiome-based ML models have been developed for other applications, including age prediction (Huang *et al.* 2020; Chen *et al.* 2022). ML models based on random forest regression revealed different levels of accuracy using human skin, oral, and gut microbiomes, with the model using the skin microbiome achieving the most accurate age prediction (Huang *et al.* 2020). A multi-view learning based model was developed for age prediction using compositional and functional features of microbiome (Chen *et al.* 2022). A model was built for predicting body mass index (BMI) using microbiome data (Liang *et al.* 2023), achieving BMI predictions with a mean absolute error (MAE) of about 2 $kg/m^{2}$. The human microbiome may be used as a resource in the forensics toolkit (Clarke *et al.* 2017), as human-associated bacterial DNA can be used to uniquely identify an individual (Franzosa *et al.* 2015), and even to provide information about their life and behavioral patterns (Kort *et al.* 2014).

Considering that microbiome composition reflects the impacts of many factors on the microbiota, and is associated with host phenotypes, here we propose a unified predictive model for the various metadata and human diseases. Although our focus is on microbiome-based disease prediction, our model also predicts the metadata including age, gender, BMI, and body site if they are missing. We showed that by doing this, we can not only improve the accuracy of disease prediction, but also improve the generalizability of the predictive model. We called our new model MicroKPNN-MT as it incorporates prior-knowledge as in MicroKPNN (Monshizadeh and Ye 2024). In addition, MicroKPNN-MT is a multitask and multiclass classification model: it includes individual decoders for metadata predictions, and the decoder for disease prediction is multiclass. Since most metagenomics projects studying microbiome-disease association often have healthy samples (control), our multiclass classification model enables the utilization of all these healthy samples from different projects. We applied our model to the mBodyMap dataset (Jin *et al.* 2022), covering 25 different human diseases, and demonstrated its potential as a predictive tool for multiple diseases and identifying microbial markers for the diseases and other metadata such as age.

## 2 Materials and methods

### 2.1 Human microbiome data

We used the mBodyMap database (Jin *et al.* 2022), which offers a comprehensive collection of human metagenomic data and their species abundance profiles derived using state-of-the-art tools. Reads processing and taxonomic assignments for all the datasets included in mBodyMap were done using the same set of tools (Jin *et al.* 2022). For taxonomic assignments, MAPseq (v1.2) (Matias Rodrigues *et al.* 2017) was used for 16S rRNA sequencing data, and MetaPhlAn2 (Segata *et al.* 2012) (default parameters) was used for shotgun metagenomic sequences. The database also boasts a carefully curated set of human-related metadata, including information on diseases and health.

We excluded the samples with the sum of relative abundances < 90 (%) and excluded diseases that had < 50 samples. We ended up with 34,233 samples from 56 projects, involving 25 diseases. A total of 6,052 species were identified from these samples. All samples have body site information, whereas other metadata have various levels of incompleteness: 6422 samples have age information, 23 804 samples have gender information, and only 2094 samples have BMI details.

**Figure 1.**
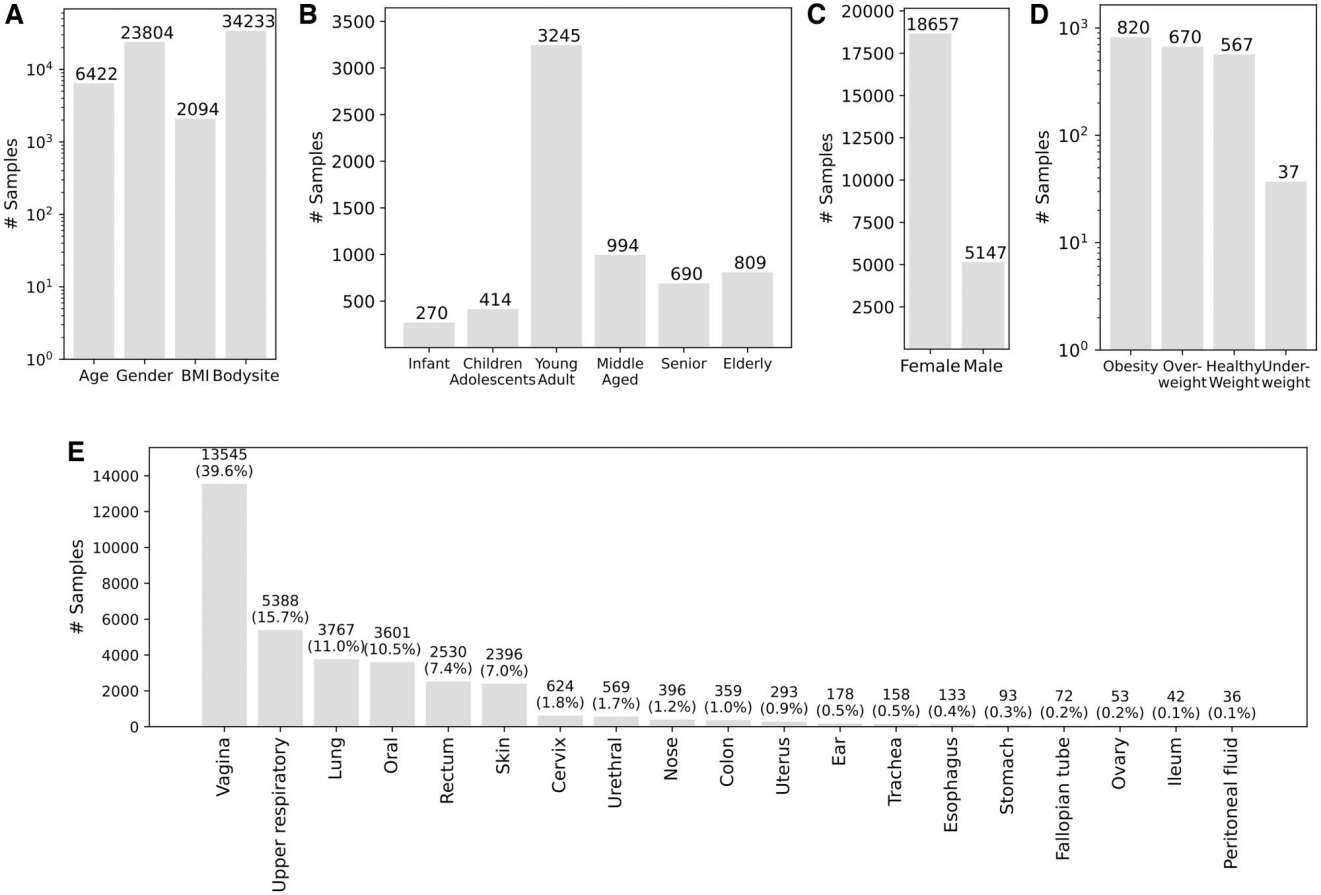
Breakdown of the samples according to the metadata. (A) The number of available samples for each metadata. (B) The number of samples within each age category. (C) The number of samples for each gender. (D) The number of samples in each BMI category. (E) The number (percentage) of samples collected from various body sites.

Our experiments showed that categorized age and BMI worked better for our application compared to their actual values. So, we performed preprocessing on the collected data. Specifically, we categorized BMI data qualitatively based on standard definitions: underweight (BMI < 18.5), healthy weight (18.5 ≤ BMI < 25), overweight (25 ≤ BMI < 30), and obesity (BMI ≥ 30). Additionally, age data were grouped into qualitative categories including infant (age ≤ 3), children adolescents (3 < age ≤ 18), young adult (18 < age ≤ 35), middle aged (35 < age ≤ 50), senior (50 < age ≤ 65), and elderly (age > 65). See Fig. 1 for the breakdown of the samples according to the different metadata.

### 2.2 The model architecture

**Figure 2.**
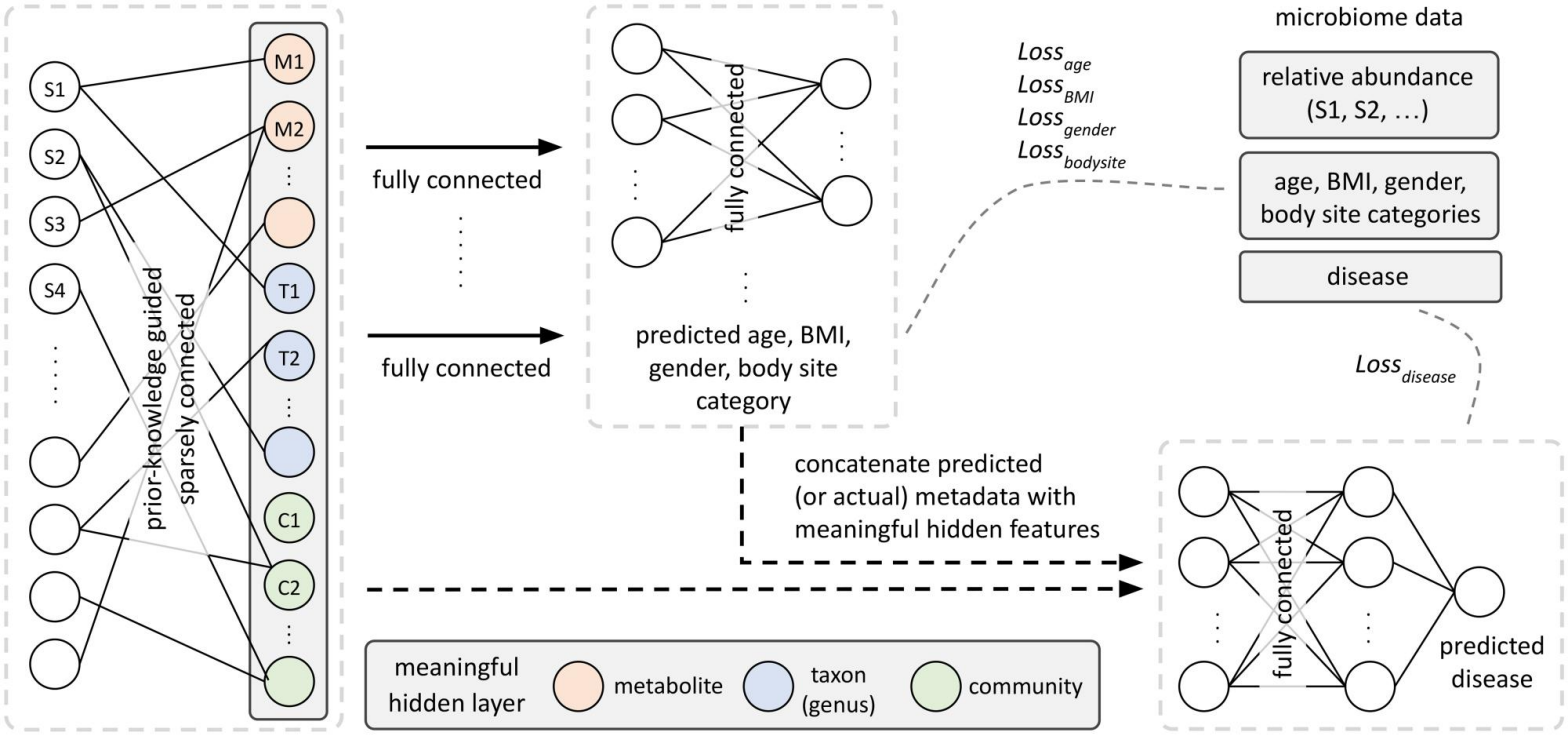
Model architecture used in MicroKPNN-MT for multitask and multiclass classification. The MicroKPNN-MT model is designed to predict human phenotype and infer missing metadata, such as BMI, age, and gender, based on microbiome data. This architecture combines microbiome input with knowledge-primed layers, which incorporate prior biological information to improve prediction accuracy and interpretability. The multitask structure allows the model to generate phenotype predictions while simultaneously predicting missing metadata. By utilizing available metadata or inferring it when missing, the model addresses confounding variables and enhances the generalizability of microbiome-based predictions. This approach also enables the identification of microbial markers potentially influencing host phenotypes, supporting both predictive accuracy and biological insight.

In this study, we propose a novel neural network architecture, termed MicroKPNN-MT, designed to capture complex relationships within human microbiome samples. The architecture is tailored to incorporate prior knowledge about microbial interactions, taxonomic relationships, and community structures for microbiome-based human disease prediction. Furthermore, MicroKPNN-MT utilizes metadata (age, gender, BMI, and body site) to enhance disease prediction. For the samples with missing metadata, MicroKPNN-MT predicts the missing metadata using additional decoders in the model, and predicted metadata are used for disease prediction. Figure 2 shows the overall model architecture, which comprises several key components outlined below.

#### 2.2.1 Input layer

The input layer of the neural network is composed of species abundance data derived from human microbiome samples. Each input node represents the relative abundance of a specific microbial species in the sample. We used the species abundance data provided by mBodyMap (Jin *et al.* 2022).

#### 2.2.2 Customized linear layer (MaskedLinear)

To leverage prior knowledge effectively, the first hidden layer employs a customized linear layer, denoted as MaskedLinear. This layer enforces a mask on the connections between the input and hidden layers based on prior knowledge. The mask is designed to capture relationships among three distinct groups: metabolites, taxa, and communities.

- Metabolites (pink nodes in Figure 2). Each metabolite is represented by two nodes in the hidden layer: one for production and one for consumption. Edges connect producer species in the input layer to the production node and consumer species to the consumption node. The metabolic edges are created according to the NJS16 metabolic network (Sung *et al.* 2017). There are a total of 281 different metabolites.
- Taxa (blue nodes). Taxonomic relationships are encoded using the NCBI hierarchical taxonomy, which we obtained by downloading the files from the NCBI FTP server through the NCBI Taxonomy database web page in December 2023. Edges connect species in the input layer with their corresponding genus. We note that MicroKPNN could exploit different taxonomic ranks in this hidden layer (genus, order, etc). MicroKPNN-MT uses the genus as the taxonomic rank, as it significantly reduces the time complexity by eliminating the search for taxonomic ranks, and also in MicroKPNN, we showed that using the genus generally gave good results across different datasets and diseases.
- Communities (green nodes). Each node represents a community, and all species belonging to the community have an edge connecting to the node. Communities are inferred from a species co-occurrence network using advanced network inference approaches that can handle sparse compositional data (Lam and Ye 2022). The co-occurrence network was constructed based on compositional data of more than 4000 human microbiome datasets, and the Leiden algorithm was applied to detect communities of microbial species in the network.

#### 2.2.3 Metadata decoders

A dedicated decoder is included for each metadata (age, gender, BMI, and body site). Each decoder comprises one fully connected layer with dimensions matching the MaskedLinear layer. The output dimensions of these decoders align with the number of classes in each metadata, except for gender, which has a single output with a sigmoid activation function.

#### 2.2.4 Disease decoder

An additional decoder is introduced for disease prediction, taking input from the output of the metadata decoders and the customized layer. This decoder includes one fully connected layer with dimensions matching the MaskedLinear layer. The output dimension matches the number of classes in the disease prediction task.

To enhance disease prediction accuracy, metadata are masked in the model. If true values for metadata are available, they are used directly. Otherwise predicted metadata from the metadata encoders are utilized for disease prediction.

### 2.3 Model training and performance evaluation

We adopted a five-fold cross-validation strategy to evaluate the performance of our model. Special care was taken to ensure that each fold maintained a consistent distribution of diseases, enhancing the reliability of our results.

To balance the effects of metadata that have different numbers of available samples, we use weighted cross-entropy and binary cross-entropy as the loss function:

$$L=\frac{n}{n_{\text{age}}}\sum_{i=0}^{n_{\text{age}}}CE({\hat{y}}_{\text{age}},y_{\text{age}})$$

 

$$+\frac{n}{n_{\text{gender}}}\sum_{i=0}^{n_{\text{gender}}}BCE({\hat{y}}_{\text{gender}},y_{\text{gender}})$$

 

$$+\frac{n}{n_{\text{bmi}}}\sum_{i=0}^{n_{\text{bmi}}}CE({\hat{y}}_{\text{bmi}},y_{\text{bmi}})$$

 

$$+\frac{n}{n_{\text{body}\;\text{site}}}\sum_{i=0}^{n_{\text{body}\;\text{site}}}CE({\hat{y}}_{\text{body}\;\text{site}},y_{\text{body}\;\text{site}})$$

 

(1) $$+\sum_{i=0}^{n}CE({\hat{y}}_{\text{disease}},y_{\text{disease}})$$

where n, $n_{\mathrm{age}}$, $n_{\mathrm{gender}}$, etc., denote the sample counts for the entire dataset, age, gender, etc., respectively; y is the actual category, $\hat{y}$ is predicted category; *CE* and *BCE* represent cross-entropy and binary cross-entropy losses, respectively.

The performance of the model was assessed using the following metrics: accuracy (ACC), which is the fraction of correct predictions; area under the curve (AUC), an aggregated measure of the model’s performance across various decision thresholds; F1 score, which is the harmonic mean of the precision (fraction of true positives among all predicted positives) and recall scores (fraction of predicted true positives among all true positives); and the area under the precision–recall curve (AUPRC), which captures the trade-off between precision and recall, particularly valuable in assessing performance on imbalanced datasets. As our model is multiclass involving 25 diseases and healthy individuals for phenotype prediction, the metrics were computed for each phenotype, then averaged to provide the overall scores. The same approach was used for the evaluation of the metadata prediction.

These metrics were chosen to account for the imbalanced nature of the dataset we used, providing a comprehensive assessment of the model’s performance across individual diseases as well as on average.

### 2.4 Interpretation of the models

We implemented local interpretability methods to analyze individual samples and subsequently aggregated these interpretations into global ones with a particular focus on the metadata-related architecture within MicroKPNN-MT. Specifically, to measure the influence of various metadata elements on disease prediction and the impact of specific hidden nodes on metadata prediction, we employed the Integrated Gradients (Sundararajan *et al.* 2017) and Layer Conductance (Dhamdhere *et al.* 2019), respectively.

Integrated Gradients (IG) attribute NN predictions to input features, while Layer Conductance (LC) delves into individual neuron and layer contributions. IG and LC attributes, positive or negative, reflect a feature’s influence on the prediction. In our experiments, we transform these attributes into importance scores by taking their absolute values, where a higher score indicates a larger impact of a feature on a specific prediction. During the aggregation phase, to determine the overall importance of features to a specific task, we average the scores across all classes within that task. In addition, the model is trained 20 times with randomly initialized weights for a stable and credible interpretation. This approach enables a thorough interpretability analysis of MicroKPNN-MT, exploring both the contributions of the nodes to metadata prediction and the impacts of metadata on disease prediction.

### 2.5 Baseline models

We compared our model with a few baseline models including Support Vector Machine (SVM), Random Forest (RF), and eXtreme Gradient Boosting (XGBoost) for disease and metadata predictions through five-fold cross-validation. We empirically determined the optimal hyperparameters for baseline models through experimental evaluation. The SVM model employed a non-linear Gaussian Radial Basis Function (RBF) kernel with a regularization parameter of 1.0. The RF model comprised 500 decision trees. The XGBoost model utilized 500 boosting stages, with each regression estimator having a maximum depth of 5. All other hyperparameters were set to their default values as implemented in the scikit-learn package.

### 2.6 Implementation and availability

We developed MicroKPNN-MT using PyTorch, and for interpretability analysis, we employed Captum (Kokhlikyan *et al.* 2020), a package specifically designed for model interpretation within the PyTorch framework. We utilized AdamW optimizer with early stopping to mitigate overfitting. The learning rate is set to 0.001, and the batch size is set to 16. The source code of MicroKPNN-MT is available at: https://github.com/mgtools/MicroKPNN-MT. Benchmarking scripts are also made available under the same repository.

## 3 Results

### 3.1 Using metadata helps improve the disease prediction

We conducted a comprehensive comparison between disease prediction models with and without metadata integration. The primary objective was to assess the impact of incorporating metadata on the predictive performance of the models. Despite the consistent structure maintained across all models, the crucial distinction lies in the meaningful interpretation of the metadata nodes within each model.

The results (see Table 1) show a notable improvement in disease prediction outcomes when all metadata were included in the models, with the F1 score improved from 0.764 to 0.810. We also built different models incorporating only one metadata at a time to test the impact of individual metadata on the prediction. The results showed that incorporating actual or predicted metadata, especially age and body site information helped improve disease prediction (see Table 1).

**Table 1.** Comparison of the accuracy of disease prediction with and without using metadata.

Model	Accuracy (std)	AUC (std)	F1 score (std)	AUPRC (std)
disease only	0.913 (0.00274)	0.965 (0.048)	0.764 (0.203)	0.519 (0.296)
disease + age	0.917 (0.00166)	0.967 (0.049)	0.779 (0.185)	0.573 (0.290)
disease + gender	0.911 (0.00337)	0.968 (0.044)	0.760 (0.199)	0.563 (0.273)
disease + BMI	0.911 (0.00224)	0.964 (0.048)	0.760 (0.198)	0.523 (0.299)
disease + body site	0.919 (0.00241)	0.967 (0.049)	0.796 (0.170)	0.544 (0.287)
disease + all metadata	**0.924 (0.00266)**	**0.968 (0.049)**	**0.810 (0.178)**	**0.620 (0.289)**

The best performances for each evaluation metric are highlighted in bold.

**Figure 3.**
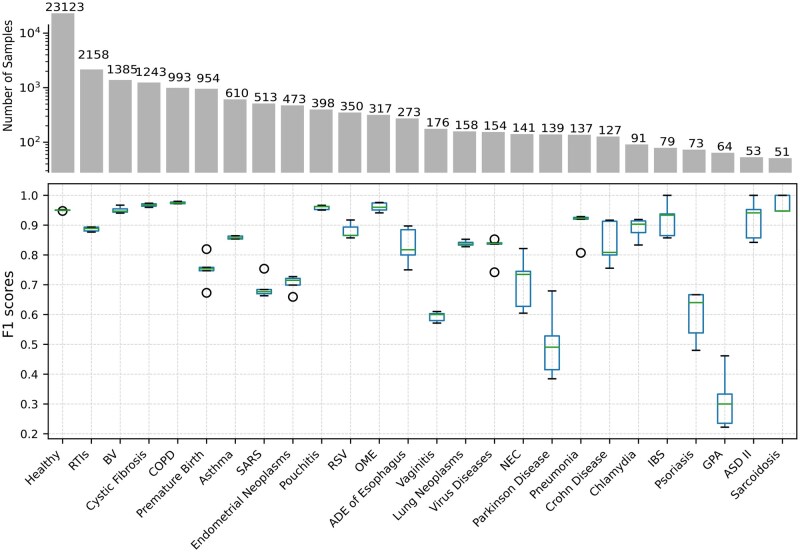
Prediction accuracy for each of the phenotypes (25 diseases + healthy) in F1 scores. The boxes with whiskers show the distribution of the F1 scores (mean and variance computed from the 5-fold cross-validations). The bars on the top show the number of available samples for each phenotype for training and testing. Abbreviations for the diseases: RTIs (respiratory tract infections), BV (bacterial vaginosis), COPD (chronic obstructive pulmonary disease), OME (otitis media with effusion), NEC (necrotizing enterocolitis), IBS (irritable bowel syndrome), GPA (granulomatosis with polyangiitis).

Figure 3 shows that different diseases have a wide range of prediction accuracy. MicroKPNN-MT predicted some diseases including cystic fibrosis with high accuracy. Fewer samples for training probably contributed to the poorer performance of some of the phenotypes including Parkinson disease, Psoriasis, and granulomatosis with polyangiitis (GPA). On the other hand, it also suggests these diseases may involve more complicated factors, and using microbiome data alone was not sufficient for accurate predictions.

A benchmark on the mBodyMap database for disease prediction is established as shown in Table 2. We compared our model with a few baseline models for all disease predictions through 5-fold cross-validation. Our model outperforms the others in terms of Accuracy (ACC) and F1 score, reflecting its effectiveness at specific decision thresholds crucial for predicting host phenotypes based on microbiome data. While RF achieves the highest AUPRC, indicating better overall precision-recall performance across all thresholds, the F1 score is more relevant for our task as it highlights the model’s performance at the critical decision points. In terms of AUC score, RF, XGBoost, and our model perform similarly, with XGBoost slightly outperforming the other two models. It is worth noting that even though the inference time of our model is not the shortest, it predicts not only diseases but also all other metadata during inference.

**Table 2.** Performance for SVM, RF, XGBoost, and ours on the disease task.

Model	ACC	AUC	F1	AUPRC	# parameters	Inference time (s)
SVM	0.808 (0.003)	0.969 (0.002)	0.503 (0.006)	0.582 (0.015)	80 M	856.122 (76.979)
RF	0.908 (0.002)	0.996 (0.010)	0.643 (0.345)	**0.881 (0.165)**	10 M	0.609 (0.050)
XGBoost	0.916 (0.003)	**0.996 (0.009)**	0.773 (0.190)	0.876 (0.152)	30 M	0.707 (0.292)
Ours (disease + all metadata)	**0.924 (0.003)**	0.968 (0.049)	**0.810 (0.178)**	0.620 (0.289)	39 M	140.400 (12.876)

All the ACC, AUC, F1, and AUPRC scores and running times are averaged across 5 folds, formatted as mean (std). The unit for the parameter number is count, and M stands for million. The best performances for each evaluation metric are highlighted in bold.

### 3.2 Missing metadata can be predicted from microbiome data

As summarized in Table 3, all metadata can be predicted using microbiome data with promising accuracy (in ACC and F1-score). Evaluations using additional metrics (AUC and AUPRC) are summarized in Supplemental Tables S1 and S2. For each metadata, we used predictions from two different models, one for disease prediction and all metadata (disease + all metadata), and the other one for disease prediction and the targeted metadata (disease + one metadata). The results show that these two models gave very similar results, although the disease + one metadata model gave marginally better results. We note age has six categories (from infant to elderly), BMI has four, sex has two, and there are 19 different body sites. BMI prediction has the lowest accuracy of about 0.592 (still significantly better than random guesses involving four choices). We attribute the poor BMI prediction to the small training data (only 6% of the samples have BMI information), among other possible reasons. We also trained and evaluated SVM, RF, and XGBoost models for metadata prediction as baselines. Since these models are not designed for multitask prediction, we trained individual models for each task. They utilized the same hyperparameters as those used for disease prediction demonstrated in the above section. Compared to these baseline models for individual tasks, our multitask models still show the highest or close to the highest performance. Our models gave the most accurate predictions of age, BMI and body site. In particular, our models outperformed the baseline models for age and BMI predictions with a large margin. For gender prediction, our models and RF outperformed other approaches, with RF outperformed our models slightly.

**Table 3.** Summary of the metadata prediction in ACC and F1-score (std).

	Age		Gender		BMI		Body site	
Model	ACC	F1	ACC	F1	ACC	F1	ACC	F1
disease + one metadata	**0.763 (<0.01)**	**0.684 (0.136)**	0.933 (<0.01)	0.901 (0.055)	**0.592 (0.019)**	**0.480 (0.211)**	0.936 (<0.01)	**0.647 (0.332)**
disease + all metadata	0.761 (<0.01)	**0.684 (0.137)**	0.931 (<0.01)	0.899 (0.057)	0.584 (0.026)	0.465 (0.225)	0.934 (<0.01)	0.633 (0.343)
SVM	0.366 (0.013)	0.355 (0.006)	0.903 (0.004)	0.774 (0.008)	0.253 (0.023)	0.247 (0.037)	0.896 (0.003)	0.533 (0.007)
RF	0.632 (0.008)	0.461 (0.235)	**0.937 (0.004)**	**0.909 (0.051)**	0.406 (0.020)	0.363 (0.194)	0.943 (0.003)	0.556 (0.405)
XGBoost	0.611 (0.009)	0.494 (0.214)	0.900 (0.005)	0.865 (0.069)	0.386 (0.031)	0.346 (0.180)	**0.944 (0.003)**	0.638 (0.352)

The best performances for each evaluation metric are highlighted in bold. Evaluations in additional metrics including AUC and AUPRC are shown in Supplemental Tables S1 and S2.

**Figure 4.**
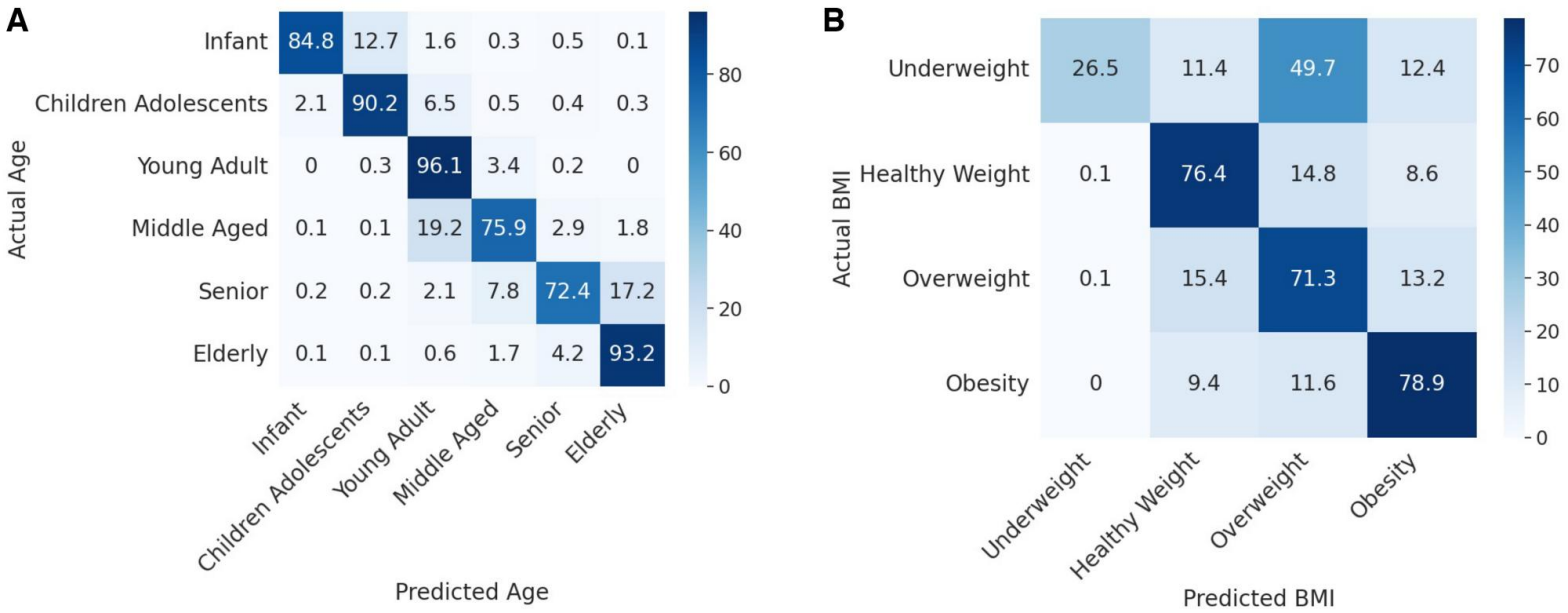
Confusion matrices summarizing the predictions of age and BMI, respectively. The percentage of samples in each cell was averaged over five-fold cross-validations. The percentages in each row add up to one. Using age prediction as an example, among the microbiomes derived from infants, 84.8% of the samples were correctly predicted as from infants, while 12.7% of the samples were predicted to be from children adolescents, and so on. See Supplemental Figure S1 for confusion matrices that show the counts of samples in each cell.

Figure 4 shows the confusion matrices for the predictions of age and BMI, showing that for most samples, their age and BMI can be correctly predicted; however, significant confusion especially between neighboring categories (e.g., elderly and senior, young adult and middle aged, healthy weight and overweight) was observed.

### 3.3 Incorporating metadata helps improve the model generalizability

To assess the robustness and generalizability of our model, we designed another experiment so that predictive models were applied to unseen data from projects that were not included in the training. We selected three diseases (out of 25 diseases) that have data from three or more projects. Additionally, we included a set of healthy samples, expanding the disease prediction task to involve four classes, namely cystic fibrosis, chronic obstructive pulmonary disease, bacterial vaginosis, and the healthy class.

We divided our dataset into two main subsets: a training dataset and a test dataset. The training dataset comprised samples from 10 projects, totaling 4380 samples. For the test dataset, we selected a different set of six projects (no overlaps between projects for train and projects for testing) for a total of 869 samples (see Supplemental Table S3 for the list of projects). This partitioning strategy aimed to provide a comprehensive evaluation of our model’s performance on a diverse range of projects and diseases. We trained and compared two models: one that utilized metadata and another that did not include this additional information.

We examined the performance of the models on the training and test datasets, respectively (see Table 4). On the training dataset, all models achieved high accuracy (RF slightly outperformed other models) with F1 score >0.96, except SVM which had F1 score of 0.856. In addition, the model using metadata yielded a slightly improved performance, highlighting the potential benefits of incorporating additional contextual information even in familiar data scenarios. On the test dataset, all models had worse performance (e.g., RF’s F1 score dropped from 0.998 to 0.674). However, what is encouraging is that our model incorporating metadata still gave reasonable predictions with F1 score of 0.856, drastically outperforming the counterpart that lacked this additional information (F1 score = 0.695). This comparison showcases the superior generalizability of our model that incorporates metadata on previously unseen data. It suggests that the integration of metadata plays a pivotal role in bolstering the model’s predictive capacity, particularly in scenarios where it encounters diverse and unfamiliar data. However, the results suggest there is still room for further improvement for generalization, as we saw a significant performance degradation on the unseen samples, despite that metadata helped.

**Table 4.** Comparison of the accuracy of disease prediction with and without using metadata on samples from unseen projects (not used for training).

Model	Accuracy (std)	AUC (std)	F1 score (std)	AUPRC (std)
SVM (train)	0.829 (0.014)	0.962 (0.005)	0.856 (0.014)	0.913 (0.013)
RF (train)	**0.997 (0.002)**	**0.999 (0.001)**	**0.998 (0.002)**	**0.999 (0.001)**
XGBoost (train)	0.996 (0.002)	**0.999 (0.001)**	0.996 (0.002)	**0.999 (0.001)**
disease only (train)	0.983 (0.005)	0.987 (0.016)	0.986 (0.012)	0.962 (0.047)
disease + all metadata (train)	0.991 (0.001)	0.994 (0.012)	0.993 (0.006)	0.988 (0.019)
SVM (test)	0.741 (0.009)	0.937 (0.004)	0.700 (0.010)	0.789 (0.013)
RF (test)	0.703 (0.005)	0.917 (0.003)	0.674 (0.004)	0.748 (0.005)
XGBoost (test)	0.668 (0.012)	0.902 (0.005)	0.610 (0.024)	0.749 (0.014)
disease only (test)	0.727 (0.017)	0.895 (0.080)	0.695 (0.115)	0.734 (0.159)
disease + all metadata (test)	**0.871 (0.026)**	**0.966 (0.023)**	**0.856 (0.140)**	**0.900 (0.095)**

The best performances for each evaluation metric are highlighted in bold for the train and test, respectively.

### 3.4 Interpretation of the predictive models

**Figure 5.**
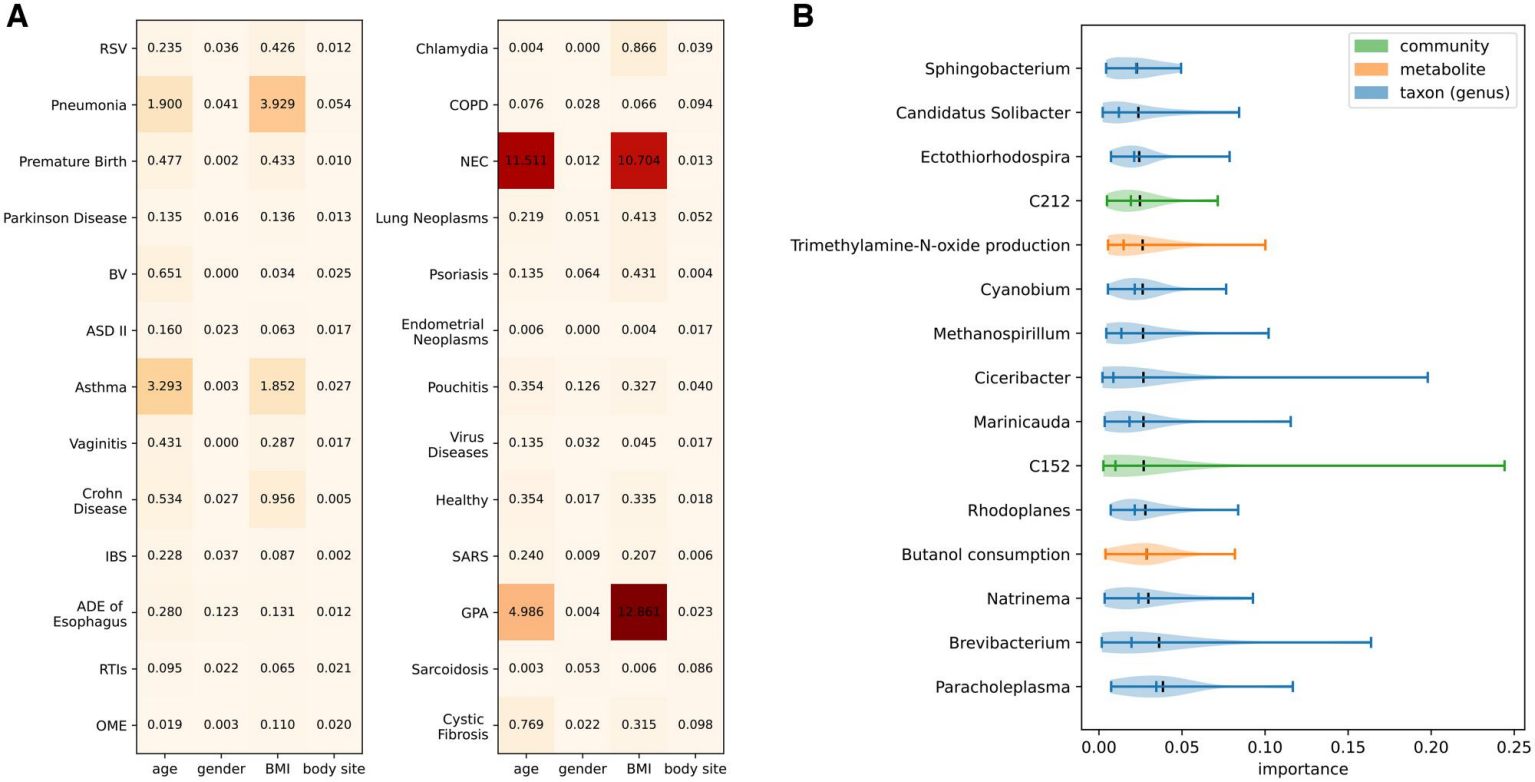
Example interpretations of the predictive models. (A) Impacts of metadata on disease prediction, where the x-axis represents metadata types, including age, gender, BMI, and body site, and the y-axis represents disease names. (B) Impacts of the meaningful hidden nodes on the age prediction, where the x-axis represents the importance scores and the y-axis lists the names of the relevant metabolites, taxa, and community nodes. The importance scores shown as numbers in (A) and represented along x-axis in (B) were computed using the Integrated Gradients and Layer Conductance approaches (see Methods). The relative values of the importance scores indicate the impacts of metadata/hidden nodes on the predictions, with higher numbers representing larger impacts.

Importance scores of the metadata and the individual nodes computed from the predictive models can be used to shed light on the impacts of metadata on disease prediction and to explain predictions. Figure 5 shows some example applications of importance scores.

Figure 5A shows the impacts of metadata on disease prediction. Most of the impact values are non-zero, suggesting that the metadata consistently enhances prediction accuracy across all diseases. Among the metadata we used, age and BMI have large impacts on the prediction of a few diseases including pneumonia, asthma, necrotizing enterocolitis (NEC), and GPA. We note that one needs to be cautious with the interpretation for GPA, as GPA was one of the diseases that were not well predicted by MicroKPNN-MT (see Figure 3). For the two respiratory diseases, the metadata have a larger impact on the prediction of asthma than COPD. We note that age and BMI showed similar patterns in their contribution to disease prediction, which could partially be caused by the correlation between age and BMI.

Figure 5B shows the impacts of the metabolite, taxon, and community nodes on age prediction. It lists the top 15 contributing hidden nodes (in the Maskedlinear layer that have biological meaning) to the age prediction, including two metabolite nodes, two community nodes, and 11 genera. Interestingly, the genus that is ranked second is *Brevibacterium*, which contains species such as *Brevibacterium epidermidis* that are typically found on the skin. This result is consistent with a previous study, which showed that using skin microbiome resulted in the most accurate prediction of age compared to microbiome from other body sites (Huang *et al.* 2020).

## 4 Discussion

We developed MicroKPNN-MT and its application to a large collection of human microbiome datasets in mBodyMap showed that our new method achieved promising results for disease predictions based on microbiome data. In addition, it provided predictions of missing metadata of the microbiome samples, which we anticipate could be utilized by other applications, considering that metadata are largely missing for the existing microbiome datasets. The comprehensive architecture we designed for MicroKPNN-MT enables the model to learn intricate relationships within microbiome samples by combining information from species abundance, prior knowledge about microbial interactions, taxonomic relationships, and community structures. The use of customized layers and metadata-specific encoders contributes to the model’s interpretability and performance across multiple prediction tasks. It is worth noting that the metadata cannot be directly integrated into the benchmark models (SVM, RF, and XGBoost) for two main reasons: a significant portion of the metadata is missing from the dataset, and the benchmark models are typically designed for single modal, which are not optimal for handling inputs from different domains (e.g., species abundance and various metadata).

Our experiments focus not only on the performance within datasets from the same project as shown in Table 1 and Table 3 but also on the generalizability of models on the data from unseen projects as shown in Table 4. Testing on samples from unseen projects showed a substantial reduction in the accuracy of the models due to the heterogeneous nature of the microbiome datasets. The observed divergence in performance between the models using and without using the metadata, especially on the unseen samples, showcased the significance of using metadata to enhance the model’s robustness beyond the confines of the training data. These results further emphasize the potential of metadata-driven approaches to improve predictive outcomes in real-world healthcare applications.

Although it was built upon MicroKPNN, MicroKPNN-MT is a very different tool for multi-class predictions and at the same time provides predictions for missing metadata. It is a unique model from these perspectives, and to our best knowledge there are no such existing tools available that does all at the same time. There were models that have been developed for example for age prediction based on microbiomes, and separate models have to be trained for different diseases, but our model can provide predictions of all metadata and all diseases currently included in the model. We also note that for comparison purpose, we reported the accuracy of the metadata prediction by traditional methods such as SVM and RF, however, unlike our method, different models have to be trained for these methods for different metadata.

We used microbiome datasets collected in mBodyMap to train and test the models. The mBodyMap dataset is comprehensive containing tens of thousands of samples, and the datasets from different projects were reanalyzed using the same procedures. Limitations include that there are other methods that can be used to infer species profiles from metagenomes, and studies have shown that the choice of bioinformatics analyses could have an impact on the utility of microbiome data (Liu *et al.* 2024).

We note that the prior-knowledge used in current MicroKPNN-MT is incomplete. For example, the community structure used in MicroKPNN-MT was inferred from gut (stool) microbiomes. Although there are significant overlaps of the microbial species found in the different body sites with those found in gut microbiome, different body sites have distinct microbial species profiles. We anticipate that such community structures can be refined when human microbiomes from different body sites are included for the co-occurrence and community detection.

We anticipate that MicroKPNN-NT can be further improved with additional optimization of the models and adding additional functionality. We observed that the model for both disease and BMI prediction actually resulted in slightly worse performance for disease prediction compared to the model that did disease prediction only (see Table 1). One direction of optimization is to try different combinations of the metadata and see how that changes the performance. Second, MicroKPNN-NT can be improved for predicting multiple diseases (a person could have disease A and disease B). Currently, MicroKPNN-MT only predicts one class for the health status prediction (healthy or one of the other 25 diseases). Finally, it is important to further develop MicroKPNN-MT so that it can provide confidence for prediction so if a microbiome dataset is derived from an individual who doesn’t have any of the included phenotypes, MicroKPNN-MT prediction should be able to reflect that.

## Supplementary Material

vbae203_Supplementary_Data

## Data Availability

The MicroKPNN-MT programs, along with benchmark scripts, are archived at Zenodo (DOI: 10.5281/zenodo.14231033).

## References

[vbae203-B1] Bannier MA , van Best N, Bervoets L et al Gut microbiota in wheezing preschool children and the association with childhood asthma. Allergy 2020;75:1473–6. 31838753 10.1111/all.14156 PMC7317729

[vbae203-B2] Bélteky M , Milletich PL, Ahrens AP et al Infant gut microbiome composition correlated with type 1 diabetes acquisition in the general population: the abis study. Diabetologia 2023;66:1116–28. 36964264 10.1007/s00125-023-05895-7

[vbae203-B3] Blekhman R , Goodrich JK, Huang K et al Host genetic variation impacts microbiome composition across human body sites. Genome Biol 2015;16:191. 26374288 10.1186/s13059-015-0759-1 PMC4570153

[vbae203-B4] Bowerman KL , Rehman SF, Vaughan A et al Disease-associated gut microbiome and metabolome changes in patients with chronic obstructive pulmonary disease. Nat Commun 2020;11:5886. 33208745 10.1038/s41467-020-19701-0 PMC7676259

[vbae203-B5] Caussy C , Tripathi A, Humphrey G et al A gut microbiome signature for cirrhosis due to nonalcoholic fatty liver disease. Nat Commun 2019;10:1406. 30926798 10.1038/s41467-019-09455-9 PMC6440960

[vbae203-B6] Chen Y , Wang H, Lu W et al Human gut microbiome aging clocks based on taxonomic and functional signatures through multi-view learning. Gut Microbes 2022;14:2025016. 35040752 10.1080/19490976.2021.2025016 PMC8773134

[vbae203-B7] Clarke TH , Gomez A, Singh H et al Integrating the microbiome as a resource in the forensics toolkit. Forensic Sci Int Genet 2017;30:141–7. 28728057 10.1016/j.fsigen.2017.06.008

[vbae203-B8] Dhamdhere K , Sundararajan M, Yan Q. How important is a neuron. In *International Conference on Learning Representations*, May 6-9, 2019, New Orleans.

[vbae203-B9] Fioravanti D , Giarratano Y, Maggio V et al Phylogenetic convolutional neural networks in metagenomics. BMC Bioinformatics 2018;19:49–13. 29536822 10.1186/s12859-018-2033-5 PMC5850953

[vbae203-B10] Fransen F , van Beek AA, Borghuis T et al The impact of gut microbiota on gender-specific differences in immunity. Front Immunol 2017;8:754. 28713378 10.3389/fimmu.2017.00754 PMC5491612

[vbae203-B11] Franzosa EA , Huang K, Meadow JF et al Identifying personal microbiomes using metagenomic codes. Proc Natl Acad Sci U S A 2015;112:E2930–E2938. 25964341 10.1073/pnas.1423854112 PMC4460507

[vbae203-B12] Hou K , Wu ZX, Chen XY et al Microbiota in health and diseases. Signal Transduct Target Ther 2022;7:135. 35461318 10.1038/s41392-022-00974-4 PMC9034083

[vbae203-B13] Huang S , Haiminen N, Carrieri AP et al Human skin, oral, and gut microbiomes predict chronological age. Msystems 2020;5:e00630–19. 32047061 10.1128/mSystems.00630-19 PMC7018528

[vbae203-B14] Huttenhower C , Gevers D, Knight R et al Structure, function and diversity of the healthy human microbiome. Nature 2012;486:207–14. 22699609 10.1038/nature11234 PMC3564958

[vbae203-B15] Iatcu CO , Steen A, Covasa M. Gut microbiota and complications of type-2 diabetes. Nutrients 2021;14:166. 35011044 10.3390/nu14010166 PMC8747253

[vbae203-B16] Jin H , Hu G, Sun C et al Mbodymap: a curated database for microbes across human body and their associations with health and diseases. Nucleic Acids Res 2022;50:D808–D816. 34718713 10.1093/nar/gkab973 PMC8728210

[vbae203-B17] Kim N. Sex difference of gut microbiota. In: Kim N (ed.) Sex/gender-specific medicine in the gastrointestinal diseases. Springer, 2022, 363–77.

[vbae203-B18] Kokhlikyan N , Miglani V, Martin M et al Captum: A unified and generic model interpretability library for pytorch, 2020.

[vbae203-B19] Kort R , Caspers M, van de Graaf A et al Shaping the oral microbiota through intimate kissing. Microbiome 2014;2:41–8. 25408893 10.1186/2049-2618-2-41 PMC4233210

[vbae203-B20] Lam TJ , Ye Y. Meta-analysis of microbiome association networks reveal patterns of dysbiosis in diseased microbiomes. Sci Rep 2022;12:17482. 36261472 10.1038/s41598-022-22541-1 PMC9581956

[vbae203-B21] Laterza L , Mignini I. The microbiome revolution: new insights for personalized medicine. J Pers Med 2022;12:1520. 36143305 10.3390/jpm12091520 PMC9503711

[vbae203-B22] Le Goallec A , Tierney B, Luber JM et al A systematic machine learning and data type comparison yields metagenomic predictors of infant age, sex, breastfeeding, antibiotic usage, country of origin, and delivery type. PLoS Comput Biol 2020;16:e1007895. 32392251 10.1371/journal.pcbi.1007895 PMC7241849

[vbae203-B23] Liang Y , Dou S, Zhao G et al Prediction of bmi traits in the chinese population based on the gut metagenome. Microb Cell Fact 2023;22:250. 38066544 10.1186/s12934-023-02255-3 PMC10704812

[vbae203-B24] Liu Y , Fachrul M, Inouye M et al Harnessing human microbiomes for disease prediction. Trends in Microbiology 2024;32:722. 38246848 10.1016/j.tim.2023.12.004

[vbae203-B25] Marcos-Zambrano LJ , Karaduzovic-Hadziabdic K, Loncar Turukalo T et al Applications of machine learning in human microbiome studies: a review on feature selection, biomarker identification, disease prediction and treatment. Front Microbiol 2021;12:313. 10.3389/fmicb.2021.634511 PMC7962872 33737920

[vbae203-B26] Matias Rodrigues JF , Schmidt T, Tackmann J et al Mapseq: highly efficient k-mer search with confidence estimates, for rrna sequence analysis. Bioinformatics 2017;33:3808–10. 28961926 10.1093/bioinformatics/btx517 PMC5860325

[vbae203-B27] Mills RH , Dulai PS, Vázquez-Baeza Y et al Multi-omics analyses of the ulcerative colitis gut microbiome link bacteroides vulgatus proteases with disease severity. Nat Microbiol 2022;7:262–76. 35087228 10.1038/s41564-021-01050-3 PMC8852248

[vbae203-B28] Monshizadeh M , Ye Y. Incorporating metabolic activity, taxonomy and community structure to improve microbiome-based predictive models for host phenotype prediction. Gut Microbes 2024;16:2302076. 38214657 10.1080/19490976.2024.2302076 PMC10793686

[vbae203-B29] Oh M , Zhang L. Deepmicro: deep representation learning for disease prediction based on microbiome data. Sci Rep 2020;10:6026–9. 32265477 10.1038/s41598-020-63159-5 PMC7138789

[vbae203-B30] Puschhof J , Elinav E. Human microbiome research: growing pains and future promises. PLoS Biol 2023;21:e3002053. 36930679 10.1371/journal.pbio.3002053 PMC10057739

[vbae203-B31] Reiman D , Metwally AA, Sun J et al Popphy-cnn: a phylogenetic tree embedded architecture for convolutional neural networks to predict host phenotype from metagenomic data. IEEE J Biomed Health Inform 2020;24:2993–3001. 32396115 10.1109/JBHI.2020.2993761

[vbae203-B32] Ruuskanen MO , Erawijantari PP, Havulinna AS et al Gut microbiome composition is predictive of incident type 2 diabetes in a population cohort of 5,572 finnish adults. Diabetes Care 2022;45:811–8. 35100347 10.2337/dc21-2358 PMC9016732

[vbae203-B33] Segata N , Waldron L, Ballarini A et al Metagenomic microbial community profiling using unique clade-specific marker genes. Nat Methods 2012;9:811–4. 22688413 10.1038/nmeth.2066 PMC3443552

[vbae203-B34] Sundararajan M , Taly A, Yan Q. Axiomatic attribution for deep networks. In *International conference on machine learning*, pp. 3319–28. PMLR, 2017.

[vbae203-B35] Sung J , Kim S, Cabatbat J et al Global metabolic interaction network of the human gut microbiota for context-specific community-scale analysis. Nat Commun 2017;8:15393–12. 28585563 10.1038/ncomms15393 PMC5467172

[vbae203-B36] Valeri F , Endres K. How biological sex of the host shapes its gut microbiota. Front Neuroendocrinol 2021;61:100912. 33713673 10.1016/j.yfrne.2021.100912

[vbae203-B37] Wilkins LJ , Monga M, Miller AW. Defining dysbiosis for a cluster of chronic diseases. Sci Rep 2019;9:12918. 31501492 10.1038/s41598-019-49452-y PMC6733864

[vbae203-B38] Wirbel J , Zych K, Essex M et al Microbiome meta-analysis and cross-disease comparison enabled by the siamcat machine learning toolbox. Genome Biol 2021;22:93–27. 33785070 10.1186/s13059-021-02306-1 PMC8008609

[vbae203-B39] Zheng P , Li Z, Zhou Z. Gut microbiome in type 1 diabetes: a comprehensive review. Diabetes Metab Res Rev 2018;34:e3043. 29929213 10.1002/dmrr.3043 PMC6220847

