# Supplementary material for: Multitask knowledge-primed neural network for predicting missing metadata and host phenotype based on human microbiome
Source: Bioinform Adv. 2024 Dec 13;5(1):vbae203. doi: 10.1093/bioadv/vbae203 (PMC11676323; doi:10.1093/bioadv/vbae203)
Supplement: vbae203_Supplementary_Data [file vbae203_supplementary_data.pdf]

# Supplemental Information: Multitask Knowledge-primed Neural Network for Predicting Missing Metadata and Host Phenotype based on Human Microbiome

Mahsa Monshizadeh[1,2], Yuhui Hong[1,2], and Yuzhen Ye[1,*]

[1]Computer Science Department, Luddy School of Informatics, Computing and Engineering, Indiana University, Bloomington, IN, USA
[2]These two authors contribute equally to the paper
[*]Corresponding author: yye@iu.edu

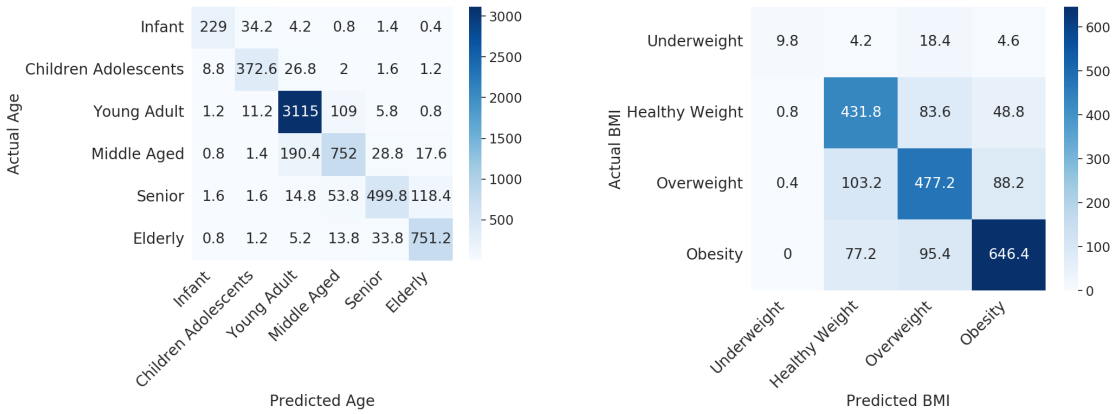


Supplemental Figure S1: Confusion matrices summarizing the predictions of age and BMI, respectively. The count of samples in each cell was averaged over 5-fold cross-validations.

Supplemental Table S1: Summary of the metadata (age and gender) prediction in ACC/AUC/F1/AUPRC score (std).

| Model | Age | | | | Gender | | | |
|---|---|---|---|---|---|---|---|---|
| | ACC | AUC | F1 | AUPRC | ACC | AUC | F1 | AUPRC |
| disease + one metadata | 0.763 (<0.01) | 0.886 (0.063) | 0.684 (0.136) | 0.571 (0.179) | 0.933 (<0.01) | 0.968 (<0.01) | 0.901 (0.055) | 0.894 (0.014) |
| disease + all metadata | 0.761 (<0.01) | 0.884 (0.068) | 0.684 (0.137) | 0.564 (0.178) | 0.931 (<0.01) | 0.966 (<0.01) | 0.899 (0.057) | 0.891 (0.010) |
| SVM | 0.366 (0.013) | 0.838 (0.008) | 0.355 (0.006) | 0.497 (0.019) | 0.903 (0.004) | 0.957 (0.003) | 0.774 (0.008) | 0.847 (0.012) |
| RF | 0.632 (0.008) | 0.938 (0.036) | 0.461 (0.235) | 0.694 (0.159) | 0.937 (0.004) | 0.985 (0.001) | 0.909 (0.051) | 0.946 (0.004) |
| XGBoost | 0.611 (0.009) | 0.909 (0.041) | 0.494 (0.214) | 0.648 (0.161) | 0.900 (0.005) | 0.966 (0.002) | 0.865 (0.069) | 0.876 (0.007) |

Supplemental Table S2: Summary of the metadata (BMI and body site) prediction in ACC/AUC/F1/AUPRC score (std).

| Model | BMI | | | | Body site | | | |
|---|---|---|---|---|---|---|---|---|
| | ACC | AUC | F1 | AUPRC | ACC | AUC | F1 | AUPRC |
| disease + one metadata | 0.592 (0.019) | 0.779 (0.074) | 0.480 (0.211) | 0.482 (0.173) | 0.936 (<0.01) | 0.947 (0.072) | 0.647 (0.332) | 0.536 (0.342) |
| disease + all metadata | 0.584 (0.026) | 0.777 (0.077) | 0.465 (0.225) | 0.498 (0.168) | 0.934 (<0.01) | 0.941 (0.081) | 0.633 (0.343) | 0.540 (0.341) |
| SVM | 0.253 (0.023) | 0.598 (0.043) | 0.247 (0.037) | 0.355 (0.029) | 0.896 (0.003) | 0.973 (0.001) | 0.533 (0.007) | 0.631 (0.011) |
| RF | 0.406 (0.020) | 0.703 (0.074) | 0.363 (0.194) | 0.454 (0.242) | 0.943 (0.003) | 0.995 (0.010) | 0.556 (0.405) | 0.741 (0.300) |
| XGBoost | 0.386 (0.031) | 0.675 (0.052) | 0.346 (0.180) | 0.421 (0.228) | 0.944 (0.003) | 0.992 (0.012) | 0.638 (0.352) | 0.710 (0.327) |

Supplemental Table S3: Summary of the projects for the generalizability experiment

| **Project ID** | **Number of samples** | **Diseases** | **Body Sites** | **Age (Number of available samples)** | **BMI (Number of available samples)** | **Gender (Number of available samples)** |
|---|---|---|---|---|---|---|
| SRP051730 | 44 | D003550 (CF) | Lung | 0 | 0 | 0 |
| SRP065627 | 45 | D006262 (healthy) | Vagina | 0 | 0 | 45 |
| SRP083112 | 91 | D003550 (CF) | Lung | 0 | 0 | 0 |
| SRP102480 | 715 | D029424 (COPD) | Lung | 690 | 686 | 0 |
| SRP115697 | 1484 | D006262 (healthy) | Vagina | 1484 | 0 | 1484 |
| SRP127076 | 428 | D003550 (CF) | Lung | 428 | 0 | 0 |
| SRP135694 | 248 | D003550 (CF) | Lung | 0 | 0 | 0 |
| SRP136124 | 99 | D029424 (COPD) | Lung | 99 | 0 | 0 |
| SRP187026 | 30 | D016585, D006262 (BV, healthy) | Vagina | 30 | 0 | 30 |
| SRP201810 | 1196 | D016585 (BV) | Vagina | 0 | 0 | 1196 |
| SRP041502 | 57 | D003550, D006262 (CF, healthy) | Lung | 0 | 0 | 0 |
| SRP043334 | 164 | D003550 (CF) | Lung | 0 | 0 | 0 |
| SRP073159 | 179 | D029424 (COPD) | Lung | 0 | 0 | 179 |
| SRP132537 | 179 | D016585 (BV, healthy) | Vagina | 0 | 0 | 179 |
| SRP165913 | 76 | D003550 (CF) | Lung | 76 | 0 | 76 |
| SRP192722 | 214 | D006262 (healthy) | Vagina | 214 | 214 | 214 |

Abbreviations of the diseases: CF for Cystic fibrosis, COPD for Chronic Obstructive Pulmonary Disease, and BV for Bacterial Vaginosis. The first 10 project belongs to the training dataset, while the last six projects belong to the test dataset.
